# An Integrated Testbed for Cooperative Perception with Heterogeneous Mobile and Static Sensors

**DOI:** 10.3390/s111211516

**Published:** 2011-12-09

**Authors:** Adrián Jiménez-González, José Ramiro Martínez-De Dios, Aníbal Ollero

**Affiliations:** Robotics Vision and Control Group, University of Sevilla, Escuela Superior de Ingenieros, c/Camino de los Descubrimientos, s/n 41092 Seville, Spain; E-Mails: adrianjg@cartuja.us.es (A.J.-G.); aollero@cartuja.us.es (A.O.)

**Keywords:** cooperating objects, wireless sensor networks, testbeds

## Abstract

Cooperation among devices with different sensing, computing and communication capabilities provides interesting possibilities in a growing number of problems and applications including domotics (domestic robotics), environmental monitoring or intelligent cities, among others. Despite the increasing interest in academic and industrial communities, experimental tools for evaluation and comparison of cooperative algorithms for such heterogeneous technologies are still very scarce. This paper presents a remote testbed with mobile robots and Wireless Sensor Networks (WSN) equipped with a set of low-cost off-the-shelf sensors, commonly used in cooperative perception research and applications, that present high degree of heterogeneity in their technology, sensed magnitudes, features, output bandwidth, interfaces and power consumption, among others. Its open and modular architecture allows tight integration and interoperability between mobile robots and WSN through a bidirectional protocol that enables full interaction. Moreover, the integration of standard tools and interfaces increases usability, allowing an easy extension to new hardware and software components and the reuse of code. Different levels of decentralization are considered, supporting from totally distributed to centralized approaches. Developed for the EU-funded Cooperating Objects Network of Excellence (CONET) and currently available at the School of Engineering of Seville (Spain), the testbed provides full remote control through the Internet. Numerous experiments have been performed, some of which are described in the paper.

## 1. Introduction

In the last years different technological fields have emerged in the broad context of embedded systems. Disciplines such as pervasive and ubiquitous computing, where objects of everyday use are endowed with sensing, computational and communication facilities, have appeared. Also, nodes in Wireless Sensor Networks (WSN) can collaborate together using *ad-hoc* network technologies to achieve a common mission of supervision of some area or some particular process using a set of low-cost sensors [1]. The high complementarities among these technologies, and others such as robotics, have facilitated their convergence in what has been called Cooperating Objects.

Cooperating Objects (CO) consist of embedded sensing and computing devices equipped with communication and, in some cases, actuation capabilities that are able to cooperate and organize themselves autonomously into networks to achieve a common task [2]. Cooperation among diverse types of CO, such as robots and WSN nodes, enhances their individual performance, providing synergies and a wide variety of possibilities. Cooperation plays a central role in many sensing and perception problems: collaboration among sensors is frequently required to reduce uncertainties or to increase the robustness of the perception. The complementarities between static and mobile sensors provide a wide range of possibilities for cooperative perception. That is the case of the cooperation between robots and static WSN. The measurements from static WSN nodes can be used to improve perception of sensors on robots, e.g., reducing the uncertainty in the localization of mobile robots in GPS-denied scenarios. Also, the mobility and the capability of carrying sensors and equipment of the robots are useful to enlarge the sensing range and accuracy of static WSN nodes. Robot-WSN cooperation has been proposed in a wide number of perception problems including surveillance [3], monitoring [4], localization [5], data retrieving [6], node deployment [7] and connectivity repairing [8], among many others. Moreover, robot-WSN cooperation is very interesting in active perception, where actuations (e.g., robot motion) are decided to optimize function costs that involve the cost of the actuation and the expected information gain after the actuation.

Cooperation among heterogeneous CO is not easy. Devices such as mobile robots and WSN nodes present high levels of diversity in its sensing, computational and communication capabilities. Sensors also have high degree of heterogeneity in technology, sensing features, output bandwidth, interfaces and power consumption, among others. However, this heterogeneity is in many cases the origin of interesting synergies in a growing number of applications. One of the main difficulties in the research on CO is the lack of suitable tools for testing and validating algorithms, techniques and applications [2]. In this sense, a good number of testbeds of heterogeneous mobile robots and of WSN have been developed. However, the number of testbeds that provide full interoperability, and thus full cooperation, between mobile and static systems with heterogeneous capabilities are still very scarce.

The paper describes a testbed for cooperative perception with sensors on mobile robots and on WSN. Its main objective is to test and validate collaborative algorithms, providing a benchmark to facilitate their comparison and assessment. The testbed comprises sensors with high level of heterogeneity including static and mobile cameras, laser range finders, GPS receivers, accelerometers, temperature sensors, light intensity sensors, microphones, among others. Moreover, it allows equanimity among these elements independently of their sensing, computing or communication capabilities. Its has been designed with an open and modular architecture which employs standard tools and abstract interfaces, increasing its usability and the reuse of code and making the addition of new hardware and software components fairly straightforward. Its architecture allows experiments with different levels of decentralization, including fully distributed and centralized experiments. As support infrastructure, the testbed includes a set of basic functionalities to release the user from programming the modules that may be unimportant in his particular experiment allowing them to concentrate on the algorithms to be tested. From the application development point of view, the proposed testbed lays in the gap between simulation and real implementation, being closer to simulation or to the real world depending on the particular experiment. It should be clear that its objective is not to validate techniques in real conditions since many environmental or scenario conditions cannot be easily replicated.

Installed in the School of Engineering of Seville (Spain), the testbed can be fully operated remotely through the Internet using a friendly Graphic User Interface. In operation since 2010, it has been used in the EU-funded Cooperating Object Network of Excellence CONET (INFSO-ICT-224053) [9] to assess techniques from academic and industrial communities.

The structure of this paper is as follows. Section 2 describes the related work. Section 3 briefly analyzes the testbed design and describes the main elements currently involved. The software architecture is presented in Section 4. Section 5 provides an insight into the testbed facilities and infrastructure, including remote access and basic functionalities. Section 6 illustrates the testbed describing some of the experiments carried out. Conclusions are drawn in the final section.

## 2. Related Work

The testbed presented in this paper has been developed to address a twofold objective. From a scientific viewpoint, it can be used as a tool to compare approaches, techniques, functionalities and algorithms in the same conditions. From an application point of view, it can be used as an intermediate step in the development of techniques before testing in the real application. The same experiment can be repeated many times with different conditions to assess its behavior. Also, the presented testbed means to serve a broad research and industrial community in which various technologies are present. There might be users interested only in WSN technology, only in mobile robotics and, the main focus of the presented testbed, researchers interested in the integration of both technologies. Attending to their level of integration and interoperability, the greater majority of the reported testbeds can be classified as: independent testbeds, which are intended to be used either in static WSN or in multi-robot systems; and partially integrated testbeds, which allows some integration but its focus is biased towards WSN or towards robotics.

In the robotics community, where problems associated to real implementation are of high importance, in most cases the following step after simulation is to test the algorithms in a real deployment. A high number of application-oriented testbeds have been developed to address perception problems such as planetary exploration [10], mapping [11], search and rescue [12], among others. Also, some robotics testbeds for general experimentation have been proposed. Thus, issues such as openness, use of standards and extensibility arise. A scalable testbed for large deployments of mobile robots is proposed in [13]. The testbed in [14] allows a wide range of coordinative and cooperative experiments. Most of these testbeds can not be operated remotely. One exception is HoTDeC [15], which is intended for networked and distributed control. In the last years a number of testbeds with Unmanned Aerial Vehicles (UAV) [16] and Unmanned Marine vehicles (UMV) [17] have been developed. RAVEN [18] combines two of these types of vehicles.

In the WSN community static testbeds are one of the most broadly used experimental tools. Despite being a relatively new technology, WSN community maintains an important number of mature testbeds and research on them is quite prolific due to remote and public access. Also, the use of wide-spread programming languages, APIs and middlewares is frequent among them. TWIST is a good example of a mature WSN heterogeneous testbed [19]. It comprises 260 nodes and allows public remote access. Its software architecture has been used in the development of other testbeds, such as WUSTL [20]. Other WSN testbeds are developed to meet particular needs or applications, losing generality but gaining efficiency. This is the case of Imote2 [21], which is focused on localization methods and WiNTER [22], on networking algorithms. Moreover, outdoors testbeds for monitoring in urban settings are under development, e.g., Harvard’s CitySense [23]. One of the latest tendencies is to federate testbeds, grouping them under a common API [9,24].

Also there are testbeds that partially integrate WSN and mobile robots. In some cases, the robots are used merely as mobility agents for repeatable or precise experiments [25], with higher accuracy than humans for this task. Their integration leads to testbeds for “Mobile sensor networks” [26] or “Mobile *ad hoc* networks—MANETS” [27]. In Mobile Emulab [28] robots are used to provide mobility to a static WSN. Users can remotely program the nodes, assign positions to the robots, run user programs and log data. Also, there are testbeds oriented to particular applications such as localization in delay-tolerant sensor networks [29]. In some other cases WSN are used merely as a distributed sensor for multi-robot experiments. In the iMouse testbed [30], detection using WSN is used to trigger multi-robot surveillance. In the micro-robotic testbed proposed in [31], the addition of WSN to simple mobile robots broadens their possibilities in cooperative control and sensing strategies. Its software architecture only allows centralized schemes.

The main general constraint of partially integrated testbeds is their lack of full interoperability. They are biased towards either WSN or robot experiments and cannot perform experiments that require tight integration. Also, the rigidity of the architecture is often an important constraint. In fact, fully integrated testbeds for WSN and mobile robots are still very scarce.

The Physically Embedded Intelligent Systems (PEIS) testbed was developed for the experimentation of ubiquitous computing [32]. PEIS-home scenario is a small apartment equipped with mobile robots, automatic appliances and embedded sensors. The software framework, developed within the project, is modular, flexible and abstracts hardware heterogeneity. ISROBOTNET [33] is a robot-WSN testbed developed within the framework of the URUS (Ubiquitous Robotics in Urban Settings) EU-funded project. The testbed is focused on urban robotics and includes algorithms for people tracking, detection of human activities and cooperative perception among static and mobile cameras. The Ubirobot (Ubiquitous Robotic) testbed integrates WSN, mobile robots, PDAs and Smartphones with Bluetooth [34]. However, no scientific publications, experiment descriptions, information on its current availability or further details on the basis of its integration have been found on Ubirobot. Although these testbeds seem to have a more general approach and all the objects have the ability to cooperate, most are designed to cover specific applications or scenarios. Moreover, these testbeds can only be operated locally.

Table 1 classifies existing testbeds according to their main features and the application towards they are focused, if any. Each row in the table corresponds to a level of interoperability: static WSN testbeds, robots testbed, partially integration of WSN and robots and highly integrated cooperating objects. Each column corresponds to a desirable feature in a testbed for Cooperating Objects as drawn from the related work. Also their objective application (if any) is pointed. Although the Ubirobot testbed [34] is classified as a highly integrated testbed, no details on their operation, experiment description or availability has been reported apart from those at the link [34].

The proposed testbed has been specifically designed to include the features shown in gray in Table 1. The testbed presented in this paper provides full interoperability between robots and WSN using an interface through which WSN nodes and robots can bidirectionally interchange data, requests and commands. The testbed is generalist: it is not focused on any applications, problems or technologies. As a result, the testbed allows performing a very wide range of experiments. It is easy to use. It can be remotely operated through a friendly GUI, allowing on-line remote programming, execution, visualization, monitoring and logging of the experiment. Also, a set of basic functionalities for non-expert users, well-documented APIs that allow code reuse and tutorials are available. To the best of our knowledge, no testbed with these characteristics has been reported.

## 3. Testbed Description

The objective of the testbed presented in this paper is to allow a wide range of experiments integrating mobile robots and Wireless Sensor Networks. Thus, the testbed should allow interoperability between heterogeneous systems, and should be flexible and extensible. From the user point of view it should be simple to use, reliable and robust, allow reuse of code and full remote control over the experiment. A survey including questionnaires to potential users (from academy and industry) was carried out to identify necessities, requirements and specifications, leading to its final design.

**Figure 1.**
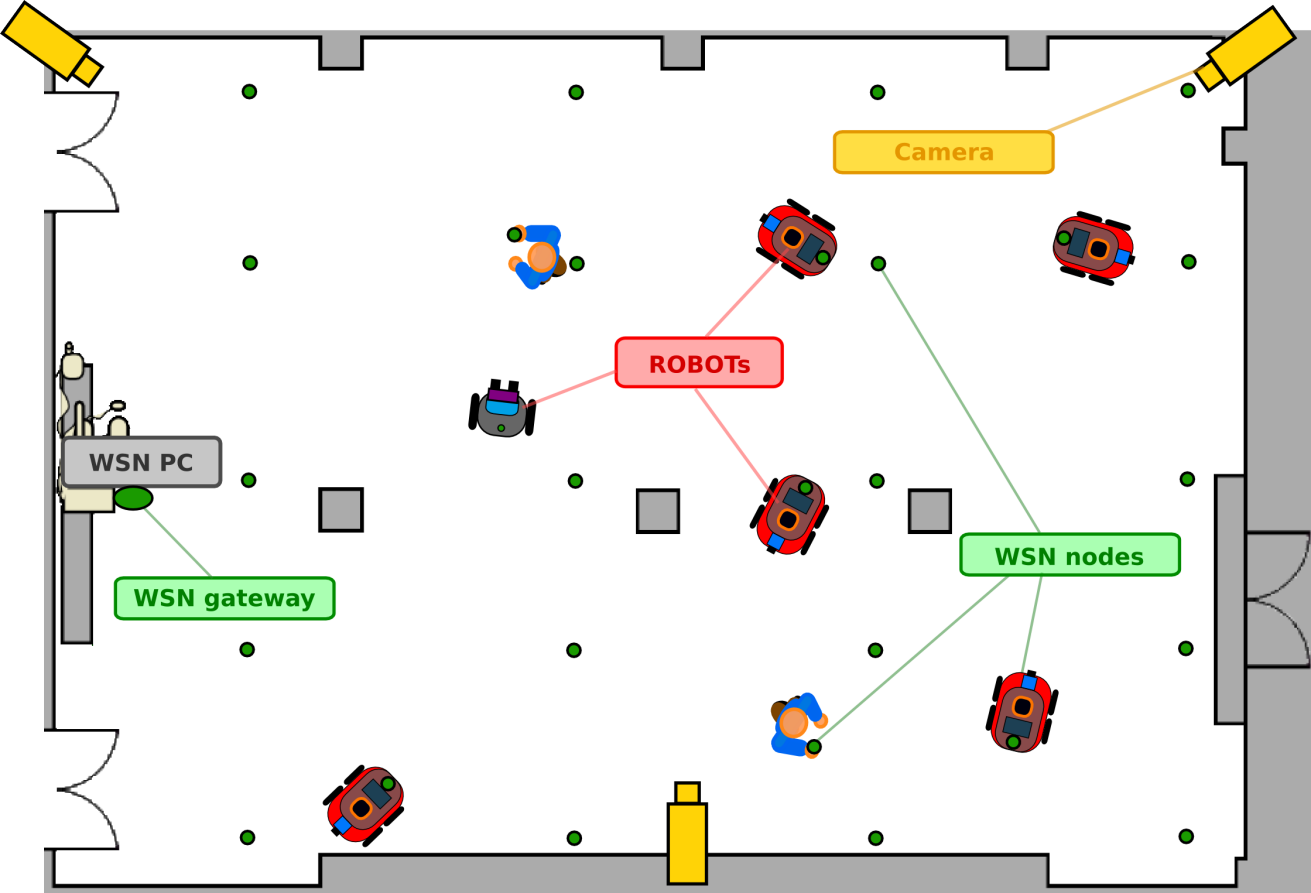
General scheme of the integrated testbed.

Figure 1 shows the basic deployment of the testbed. It is set in a room of more than 500 m^2^ (22 m × 24 m) crossed longitudinally by three columns. Two doors lead to a symmetrical room to be used in case extra space is needed. The figure shows the mobile robots and WSN nodes (green dots), that can be static or mobile, mounted on the robots or carried by people. It also includes IP cameras (in yellow) to provide remote users with general views of the experiment.

A rich variety of sensors are integrated in the testbed including cameras, laser range finders, ultrasound sensors, GPS receivers, accelerometers, temperature sensors, microphones, among others. The sensors have high degree of heterogeneity at different levels including sensed magnitudes, technology, sensing features, output bandwidth, interfaces—hardware and software—and power consumption, among others. These sensors are mounted on two different types of platforms: mobile robots and WSN. Both platforms have their own particularities. While in WSN low cost, low size and low energy constrain the features of WSN sensors, robots can carry and provide mobility to sensors with higher performance.

The following subsections present the currently available sensors, platforms and communication infrastructure. Note that the testbed modularity, flexibility and openness allow easy addition of new hardware and software components.

### 3.1. Platforms

#### Mobile Robots

**Figure 2.**
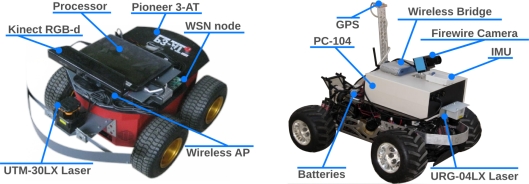
Robotic platforms and main onboard sensors used in the testbed.

The testbed currently includes 5 skid-steer holonomic Pioneer 3-AT all-terrain robots from *Mobilerobots*. Each robot is equipped with several sensors, see Figure 2 left, including one *Hokuyo* 2D laser range finder and one *Microsoft* Kinect RGB-D sensor. Each robot can also be equipped with a RGB IEEE1349 camera, in case robots with two cameras are required in an experiment. Each robot is further equipped with extra computational resources, a simple Netbook PC with an *Intel* Atom processor and 1,024 MB SDRAM, and communication equipment, a IEEE 802.11 a/b/g/n Wireless bridge. The robot in Figure 2 right, with Ackerman configuration, is also used in outdoor experiments. Based on a gas radio-controlled (RC) model, it has been enhanced with a *Hokuyo* 2D laser, a RGB IEEE1349 camera, a PC-104 with an *Intel* Pentium III processor and a Wireless a/b/g/n bridge. Each robot is equipped with one GPS card and one Inertial Measurement Unit (IMU). Besides, each robotic platform also carries one WSN node, which facilitates robot-WSN cooperation and provides extra sensing capabilities.

#### Sensor Networks

**Figure 3.**
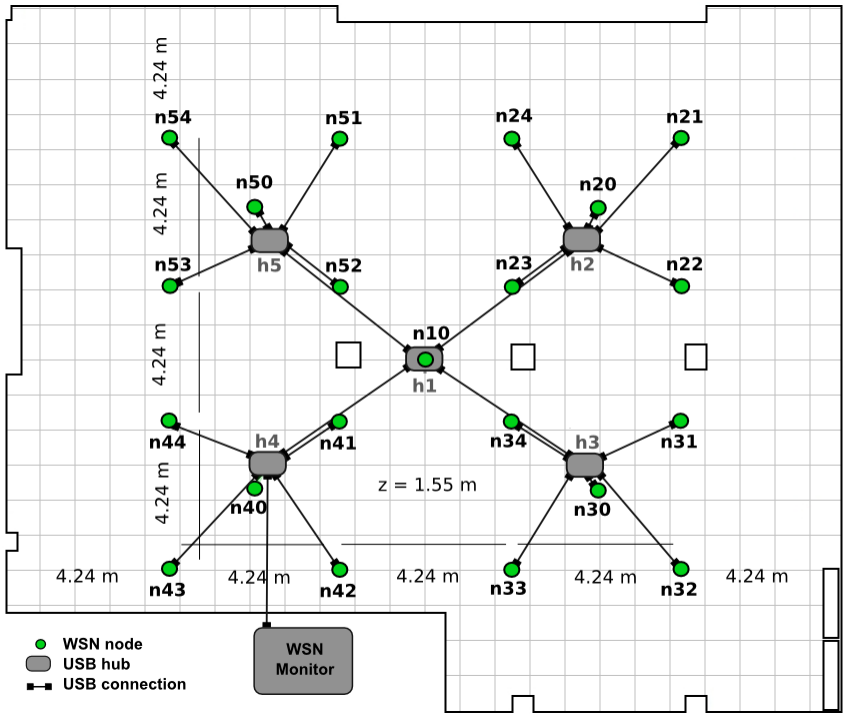
Deployment of the static WSN in the testbed room.

The testbed WSN consists of static and mobile nodes, with one node mounted on each of the robots. Since WSN nodes are powered by batteries, they can also be carried by people if it is required in the experiment. The static WSN nodes are deployed at 21 predefined locations hanging at 1.65mheight from the floor. A diagram of the deployment in the testbed room map is shown in Figure 3. This configuration allows, by switching off some nodes, establishing node clusters in order to facilitate performing WSN clustering experiments.

A dedicated processor (WSN PC) is physically connected to each single WSN node through a USB-hub tree. The WSN PC provides monitoring, reprogramming and logging capabilities and connects the WSN with the rest of the testbed elements through the Local Area Network. If required by the experiment, the WSN PC can also host more than one WSN gateways or serve as a central controller.

Four different models of WSN nodes are currently available in the testbed: TelosB, Iris, MicaZ and Mica2, all from *Crossbow*. TelosB nodes are equipped with SMD (Surface Mounted Devices) sensors whereas all other nodes need to be equipped with *Crossbow* MTS400 or MTS300 sensor boards. Also, some have been equipped with embedded cameras such as CMUcam2 and CMUcam3. A detailed description of the sensors currently available is provided in Section 3.3.

### 3.2. Communications

**Figure 4.**
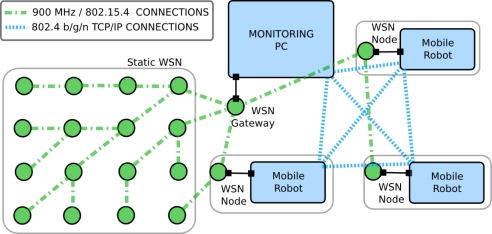
Connections among the testbed elements.

Figure 4 shows all the data connections among the testbed elements. There are two wireless networks: a wireless LAN (802.11b/g/a) that links the PC that monitors the WSN (WSN-PC) and the team of robots (represented with dashed blue lines in the figure) and the *ad-hoc* network used by the WSN nodes (represented with green lines while nodes are circles). Also, the standard Access Point based WLAN that connects the robots could be replaced by an *ad-hoc* network in case the experiment requires a more realistic communication infrastructure.

Two different WSN networks can be used in the testbed. TelosB, Iris or MicaZ nodes use IEEE 802.15.4 protocol while Mica2 nodes use an *ad-hoc* protocol that operates in the 900 MHz radio band. The IEEE 802.15.4 protocol uses the 2.4 GHz band, has a bit rate of 250 kbps and a range of less than 40 m in realistic conditions. While WSN networks were designed for low-rate and low-range communications, WiFi networks can provide up to 54/36 mbps (maximum theoretical/experimental bound) at significantly greater distances. Robot and WSN networks differ greatly in range, bandwidth, quality of service and energy consumption. Combining them allows high flexibility in routing and network combinations. In the design of the testbed, to cope with potential interference between 802.15.4 and 802.11 b/g (both use the populated 2.4 GHz band), a separate dedicated 802.11 b/g network at 5 GHz was installed and used instead of the 2.4 GHz Wifi network of the School of Engineering of Seville.

Figure 4 also shows the connections at each robot. In this configuration the robot processor is physically connected to the low-level motion controller, the Kinect, the ranger and the WSN node. The WSN nodes can be equipped with sensors but these connections are not shown in the figure for clarity.

### 3.3. Sensors

A rich variety of heterogeneous sensors are integrated in the testbed. We differentiate between robot sensors and WSN sensors due to their diverse physical characteristics, computation requirements (size and frequency of measurements) and communications needs. Table 2 schematically shows the main characteristics of the main sensors mounted on the mobile robots. Of course, although we consider them mobile sensors, we can also “make” them static by canceling the robot mobility.

Table 3 shows those corresponding to the main WSN sensors. The WSN nodes also include sensors to measure the strength of the radio signal (RSSI) interchanged among the nodes. Of course, each node model measures RSSI differently since the measurements are affected by the antenna and radio circuitry, among others. For instance, while the MicaZ uses an 1/4 wave dipole antenna with −94 dBm sensitivity, TelosB nodes use Inverted-F *μ*strip antenna with −94 dBm sensitivity. Iris nodes also uses an 1/4 wave dipole antenna but with −101 dBm sensitivity. Furthermore, some WSN nodes have also been integrated with *Figaro* gas concentration detectors (CO, CO_2_ and H_2_).

The low cost, low size and low energy consumption of WSN technology impose constraints to its sensors. WSN sensors usually have lower sensing capabilities (accuracy, sensitivity, resolution), lower output bandwidth in order to simplify the transmission and processing of the measurements and lower energy consumption than those carried by robots. The RGB cameras in the robots (21BF04) and those used by the WSN (CMUcam3) constitute a clear example of this difference. The former has a resolution of 640 × 480 pixels, a frame rate of 30 frames per second and a power consumption of 2.4 W. On the other hand, the latter provides images of 352 × 288 pixels at 115,200 bits per seconds (around 1 frame per second) but only consumes between 650 mW and 135 mW (4 to 20 times lower).

Summarizing, the testbed is equipped with a heterogeneous set of sensors that are of common use in cooperative perception research and applications. To enlarge the range of experiments all the equipment is suitable for outdoor experiments, with the exception of the Kinect distance sensor, whose operation is affected by solar light.

## 4. Software Architecture

One of the main requirements in the testbed design is the interaction of heterogeneous sensors and platforms in an open, flexible and interoperable way. Also, it should support user programs capable of executing a wide range of different algorithms and experiments. Modularity, usability, extensibility and reuse of code are also to be taken into account. The solution adopted is to use an integrating layer through which all the modules intercommunicate using standardized interfaces that abstract their particularities in APIs available for a number of programming languages.

The testbed uses Player [35] as main integrating layer. Player is an open-source middleware widely used in networked robotics research. It is based on a client/server architecture. The Server interacts with the hardware elements and uses abstract interfaces—called Player Interface—to communicate with the Player Client, which provides access to all the testbed elements through device-independent APIs. In our testbed, Player communicates, on one side, with the sensors, robots and WSN and, on the other side, with the programs developed by the users. Player includes support for a large variety of sensors, platforms and devices, making straightforward the inclusion of new elements in the testbed. Even if Player does not provide support for one specific element, its modular architecture allows simple integration by defining the Server and Client components for the new element. That was the case of Player support for WSN, which was developed during the project as will be described later. Moreover, Player is operating system independent and supports programs in several languages including C, C++, Python, Java and GNU Octave/Matlab among others. Thus, the testbed user can choose any of them to program their experiment, facilitating the programming process.

Player is one of the predecessors of ROS (Robotics Operating System) [36]. ROS provides the services that one would expect from an operating system, gaining high popularity in the robotics and other communities. The main reason not to have ROS as the testbed abstraction layer resides in its novelty: the testbed was already in operation for internal use within CONET when ROS was born. ROS is fully compatible with Player. Adaptation of the testbed architecture to ROS is object of ongoing work.

**Figure 5.**
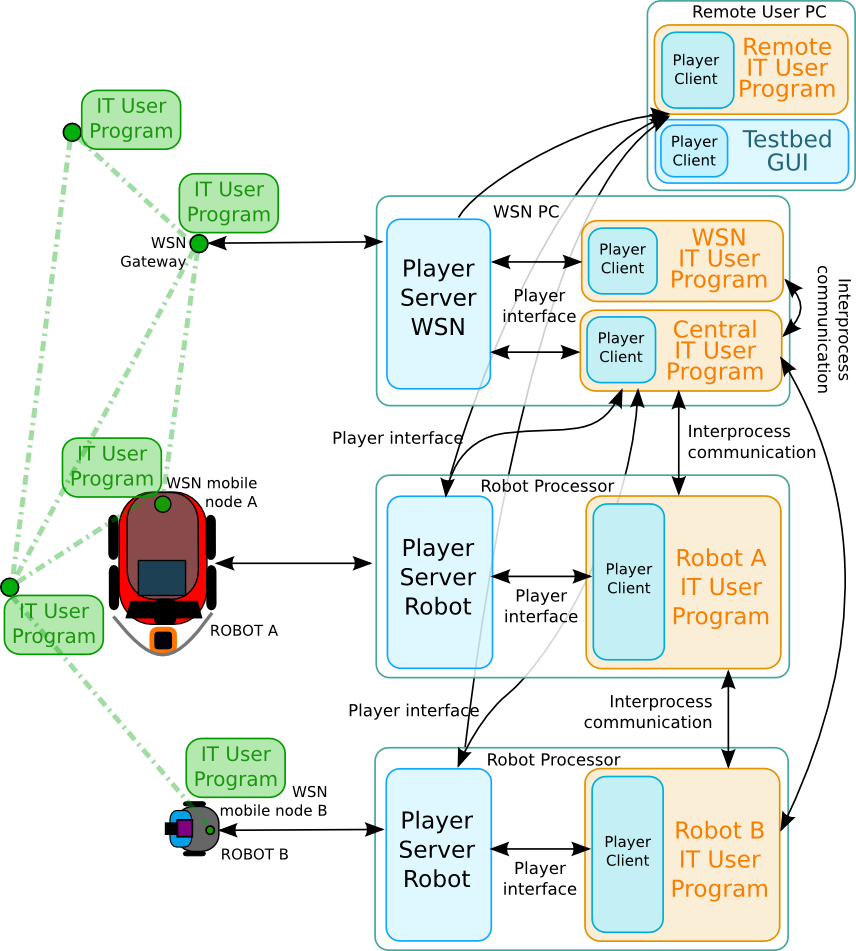
General scheme of the testbed software architecture. Player (blue squares) runs in each robot processor and in the WSN PC, connecting all elements (WSN nodes, robots and sensors). The user is allowed to program each WSN node (green square), robot, WSN PC and central controller (orange squares).

Figure 5 depicts the basic diagram of the software architecture. It shows the main processes that are running in the robot processors, the WSN nodes and the WSN PC. The Robot Servers include drivers for bidirectional communication with: the low-level robot controller, the camera, the laser and the attached WSN node. The WSN Server runs in the WSN PC, which is connected to the WSN gateway and in each of the robots to communicate with their on-board node. The WSN gateway is simply a WSN node that is connected to a PC and forwards all messages from the WSN to the PC. Note that the software architecture provided by the testbed is flexible enough to function without this element. Also various gateways could be used or even some of the mobile nodes could be gateways forwarding messages to the robots. The concrete system deployment depends on the experiment, the user needs and the code provided.

The architecture allows several degrees of centralization. In a decentralized experiment the user programs are executed on each robot and, through the Player Interfaces, they have access to the robot local sensors. Also, each Player Client can access any Player Server through a TCP/IP interprocess connection. Thus, since robots are networked, the Player Client of one robot can access the Player Server of another robot, as shown in the figure. In a centralized experiment an user Central Program can connect to all the Player Clients and have access to all the data of the experiment. Of course, scalability issues regarding bandwidth or computing resources may arise depending on the experiment. In any case, these centralized approaches can be of interest for debugging and development purposes. Also, in the figure the Central Program is running in the WSN PC. It is just an example; it could be running, for instance, in one robot processor. Following this approach, any other program required for an experiment can be included in the architecture. To have access to the hardware, it only has to connect to the corresponding Player Server and use the Player Interfaces.

Full remote access has been one of the key requirements in the design of this testbed. A Graphical User Interface (GUI) was developed to provide remote users with on-line full control of the experiment including programming, debugging, monitoring, visualization and logs management. It connects to all the Player Servers and gathers all the data of interest of the experiment. The GUI will be presented in Section 5. Several measures were adopted to prevent potential uncontrolled and malicious remote access. A Virtual Private Network (VPN) is used to secure communications through the Internet using encrypted channels based on Secure Sockets Layer (SSL), simplifying system setup and configuration. Once the users connect to the VPN server at the University of Seville, they have secure access to the testbed as if they were physically at the testbed premises. The architecture also allows user programs running remotely, at the premises of the user, as shown in the figure. They can access all the data from the experiment through the VPN. This significantly reduces the developing and debugging efforts.

Figure 5 shows with blue color the modules provided as part of the testbed infrastructure. The user should provide only the programs with the experiment he wants to carry out: robot programs, WSN programs, central programs, *etc.* The testbed also includes tools to facilitate experimentation, such as a set of commonly-used basic functionalities for robots and the WSN (that substitute the user programs) and the GUI. They will be described in Section 5.

### 4.1. Robot-WSN Integration

In the presented testbed we defined and implemented an interface that allows transparent communication between Player and the WSN independently of the internal behavior in each of them, such as operating system, messages interchanged among the nodes, node models used. The objective is to specify a common “language” between robots and WSN and, at the same time, give flexibility to allow a high number of experiments. Therefore, the user has freedom to design WSN and robot programs. This interface is used for communication between individual WSN nodes (or the WSN as a whole using a gateway) and individual robots as well as for communication between individual WSN nodes (or the WSN as a whole using a gateway) and the team of robots as a whole.

The robot-WSN interface contains three types of bidirectional messages: data messages, requests and commands, allowing a wide range of experiments. For instance, in a building safety application the robots can request the measurements from the gas concentration sensor of the WSN node they carry. Also, in WSN localization the robot can communicate its current ground-truth location to the node. Moreover, in an active perception experiment, the robot can command the WSN node to deactivate sensors when the measurements do not provide information. Furthermore, a WSN node can command the robot to move in a certain direction in order to improve its perception. Note that robots can communicate not only with the WSN node it carries, but also with any other node in the WSN. In that case the robot WSN node simply forwards the messages. Thus, the robot can request the readings from any node in the WSN and any WSN node can command any robot. For instance, in a robot-WSN data muling experiment one node could command a robot to approach a previously calculated location. Also, this robot-WSN communication can be used for sharing resources: a WSN node can send data to the robot in order to perform complex computations or to register logs benefiting from its higher processing capacities. More details on these and other experiments can be found in Section 6.

The aforementioned cooperation examples are not possible without a high degree of interaction and flexibility. Of course, similar robot-WSN cooperation approaches have been specifically developed for concrete problems, see e.g., [37]. However, they are tightly application particularized.

All the messages in the robot-WSN interface follow the same structure including a header with routing information and a body, which depends on the type of the message. Also, some application-dependent message types, for alarms, generic sensor measurements and specific sensor data such as RSSI or position were defined. Table 4 shows the format of some of these messages.

**Figure 6.**
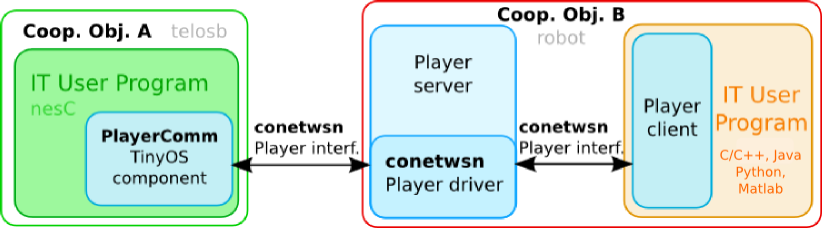
Scheme for interoperability in the testbed architecture. The testbed infrastructure (blue) abstracts hardware and interoperability specificities. The testbed user can provide code to be executed in the WSN nodes (green square) and the robots (orange square) in a variety of programming languages or use any of the basic functionalities available.

The interface was designed to allow compatibility with broadly used WSN operating systems, including TinyOS (1.x and 2.x versions) [38] and Contiki [39]. Its implementation required the development of a new Player module (*i.e.*, driver and interface). Also, a TinyOS component was developed to facilitate programs development providing a transparent API compliant with this protocol. The component was validated with *Crossbow* TelosB, Iris, MicaZ, Mica2 nodes. Other WSN nodes could be easily integrated following this interface. Figure 6 shows a diagram of the interoperability modules developed.

## 5. Users Support Infrastructure

### 5.1. Basic Commonly-Used Functionalities

The testbed was designed to carry out experiments involving only robots, experiments with only WSN nodes and experiments integrating both. In many cases a user could lack the background to be able to provide fully functional code to control all devices involved in an experiment. Also, users often may not have the time to learn the details of techniques from outside their discipline. The testbed includes a set of basic functionalities to release the user from programming the modules that may be unimportant in his particular experiment, allowing them to concentrate on the algorithms to be tested. Below are some basic functionalities currently available.

#### Indoors Positioning

Outdoors localization and orientation of mobile sensors is carried out with GPS and Inertial Measurement Units. For indoors, a beacon-based computer vision system is used. Cameras installed on the room ceiling were discarded due to the number of cameras—and processing power for their analysis—required to cover our 500 m^2^ scenario. In the solution adopted each robot is equipped with a calibrated webcam pointing at the room ceiling, on which beacons have been stuck at known locations. The beacons are distributed in a uniform square grid: the webcam always sees in the image at least one beacon square, *i.e.*, an imaginary square with one beacon at each corner. Each robot processes the images from its webcam: segments the beacons and classifies the beacons using efficient classifiers. Then, analyzing the beacon types that form the square in the image it is simple to determine unequivocally the location of the beacon square in the image. The last step is to apply homography-based techniques to determine the robot location and orientation. The method provides errors lower than 8 mm and can be executed at a suitable frame rate in the robot processors where other processes are also running.

The testbed offers the possibility to use other localization methods, such as the adaptive Monte-Carlo localization (AMCL) [40], integrated in Player. This method maintains a probability distribution of the robot poses using measurements from odometry and laser range-finders by means of a Particle Filter that adjusts its number of particles, balancing between processing speed and localization accuracy.

#### Synchronization

Sensor data synchronization is required in a wide range of experiments. In the testbed the solution adopted is to use time stamps. For robots, the well-known Network Time Protocol (NTP) is used [41]. For the WSN nodes, we implemented the Flooding Time Synchronization Protocol (FTSP) [42]. The FTSP leader node periodically sends a time synchronization message. Each node that receives the message re-sends it following a flooding strategy. The local time of each node is corrected depending on the time stamp of the message and on the sender of the message. The algorithm is efficient and obtains synchronization errors of few milliseconds, sufficient for a wide number of applications. In our case the FTSP leader node is the WSN base. The WSN PC, connected to the robots, also runs the NTP protocol. Since the WSN PC is also connected to the WSN base, the NTP is used as reference time to the FTSP leader. Thus, all robots and WSN nodes are synchronized.

#### Robot Navigation

Several robot motion control functionalities are offered including a low-level velocity control, local position control, trajectory following and random walk. Each of them includes an underlying obstacle avoidance module that ensures a certain configurable distance with the obstacle.

The random walk functionality commands the robot with pseudo-random velocity commands. Robot navigation functionalities allow the robot to follow a trajectory specified with a set of ordered waypoints. Two navigation methods are currently implemented: a global navigation path planner and a local position control module. The local position control is useful for experiments where no global localization (*i.e.*, testbed groundtruth, GPS) is available and the waypoints have to be given in robot local coordinates. Two local methods are implemented vector field histogram plus VFH+ [43], suitable for holonomic vehicles such as the Pioneer robots, and smooth nearest diagram (SND) methods [44], more suitable for Ackerman-configured vehicles, such as the testbed outdoors robot. If global robot localization is available, the global path planner decides the route from the current position to the next goal and feeds the local position controller with intermediate waypoints. The path global planning method available is the Wavefront-propagation path-planner [45].

#### WSN Data Collection

The objective is to register in the WSN PC the readings of the sensors in static and mobile WSN nodes. The static WSN nodes are programmed to periodically read from the attached sensors and send the data to the WSN gateway using the WSN routing channels. These channels are established in a prior stage called network formation. Numerous network formation methods have been proposed with the objective of minimizing the energy consumption, number of hops or optimizing robustness to failures, among others. The testbed implements the Xmesh network formation method. Xmesh is a distributed routing method based on the minimization of a cost function that considers link quality of nodes within a communication range [46]. The mobile WSN nodes attached to a robot have two alternatives to transmit their data to the WSN PC: use the robot network or use the routing channels of the WSN static network. In the first case, the messages are sent to the corresponding robot who forwards the data to the WSN PC. In the second case, the mobile node should decide the best static node, who will use the WSN routing channels. The mobile node broadcasts beacons asking for responses in order to select the static node in its radio coverage with the best link quality.

The testbed is also equipped with two WSN sniffers for network surveying. The first monitors power in every channel in the 2.4 GHz band. The second registers all packets interchanged in the WSN network.

### 5.2. Graphical User Interface

**Figure 7.**
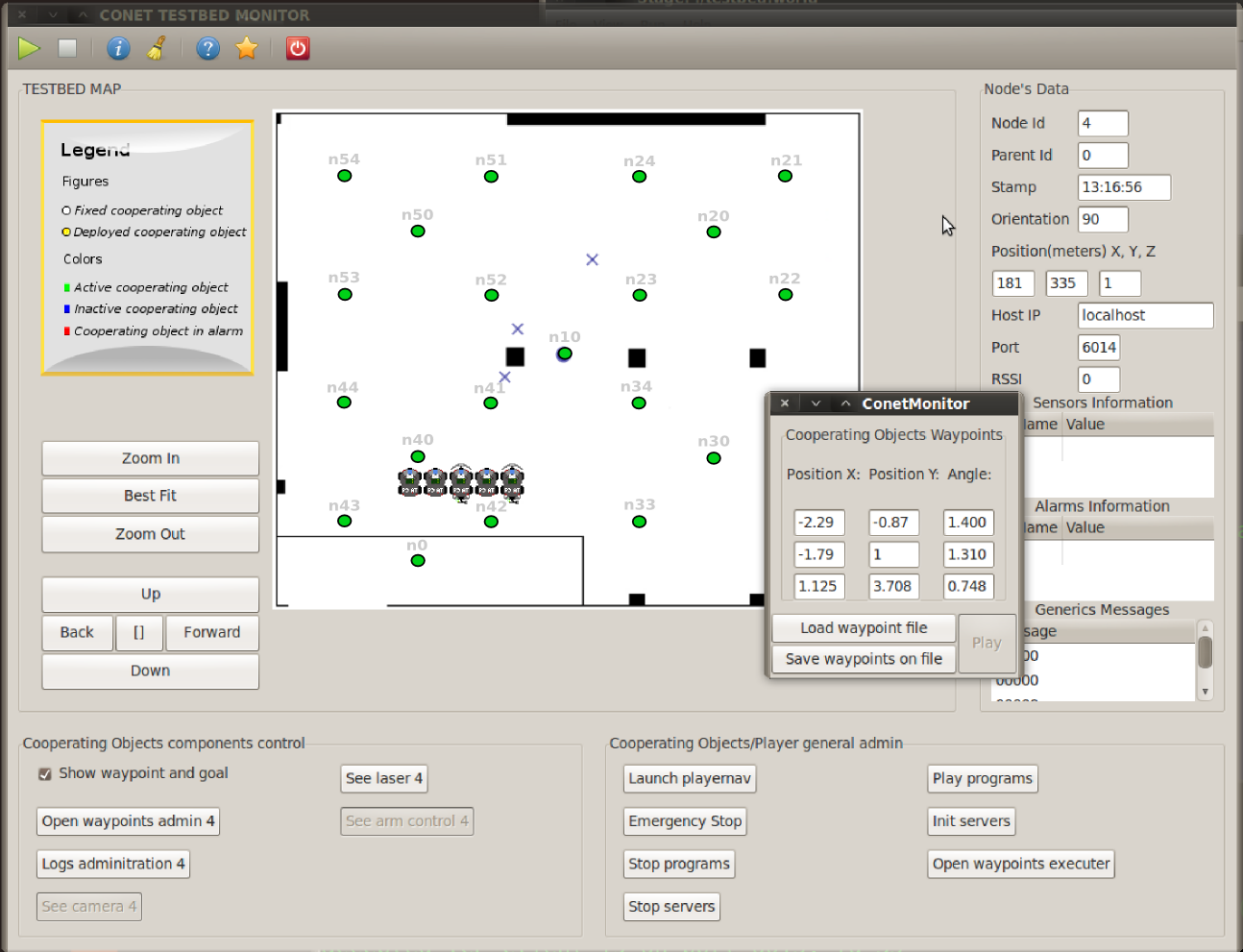
Snapshot of the testbed GUI for remote experiment control and monitoring.

The graphical user interface (GUI) in Figure 7 has been developed to facilitate the remote use of the testbed. It is fully integrated in the architecture and allows remote access to all the devices using the Player Interfaces. The GUI can be used for monitoring the experiment including the position and orientation of the robots and data from the WSN sensors. It contains tools to visualize images and laser readings from the robots. The experiment can be remotely visualized using the IP cameras as well.

The GUI also allows programming each of the elements involved in the experiment. It allows on-line configuring and running all basic functionalities for each platform. For instance, the robot trajectory following functionality can be configured by simply providing a list of waypoints. The waypoints can be given by manually writing the coordinates in the dialog box, see Figure 7, or by a simple text file. Moreover, the user can graphically, by clicking on the GUI window, define the robot waypoints. Also, if the user does not want to use the basic functionalities, the GUI allows to on-line upload user executable codes for each platform. It is also possible to on-line re-program them, in between experiments facilitating the debugging process. The GUI also allows full control of the experiment start and stop, either synchronized or on a one-by-one program basis. Finally, the GUI offers remote logging control, allowing the user to start or stop logging. To cope with potential bandwidth limitations of remote access, the user can select the data he wants to monitor and log in the GUI. Also, all experiment data are registered and logged locally and remains available to be downloaded.

The user should schedule the experiment in advance, specifying the resources involved. The testbed website [47] allows creating/editing/canceling experiments requests. The site also includes sections with datasheets of all devices, manuals and tutorials. Furthermore, it contains a download section where all necessary software, libraries and distributions are available. A Virtual Machine with all necessary software and libraries pre-installed is also available to facilitate the software download and installation process.

## 6. Experiments

A wide variety of experiments have been performed in the testbed, mostly inside the testbed room, benefiting from its controlled environment. Nonetheless, also indoors experiment outside of the controlled testbed room [48] and outdoors experiments [49] were carried out. Although some of these experiments focused on multi-robot schemes and some others on static WSN algorithms, the higher number of experiments performed concentrated on WSN-robot cooperation. In many cases this cooperation is exploited to improve perception. In others, robot-WSN collaboration is established to address other objectives, such as increasing communication robustness. This section briefly presents some of the experiments carried out trying to show an overview of the testbed capabilities.

### 6.1. Robot-WSN Collaboration

The testbed is suitable for experiments with tight WSN-robot cooperation. We describe two of them. In the first one the robot helps to improve the performance of the static WSN. In the second, the static WSN nodes guide mobile robots to reach their destination.

Network communication is often affected by connectivity boundaries and holes, or malfunctioning, mislocated or missing nodes. The objective is to diagnose a static WSN network deployed in an area and, if necessary, to repair it using robots equipped with WSN nodes. The robots deployed at certain repairing locations are used as WSN communication relays. WSN repairing could also be applied in sensing, e.g., to substitute failing nodes or to intensify the monitoring of an area. During the diagnosis step, robots surveyed the area discovering the topology of the network. The robots broadcasted beacon messages and each static nodes responded with messages containing the ID of its one-hop neighbor nodes. Each robot generated its connectivity matrix and transmitted it to the base station.

Network repairing is performed in two steps. The first one increases k-connectivity. k-connectivity expresses the number “k” of disjoint paths between any pair of nodes within the network. 0-connectivity means that there is at least one pair of disconnected nodes. An algorithm was developed to identify the minimum number of network repairing locations such that the network achieves n-connectivity. In the experiments n was taken as 1. Then, the robots were commanded to position at those locations and their WSN node becomes part of the network. The connections between the WSN nodes, and thus the improvement in the WSN connectivity, can be monitored and logged using the testbed GUI. Then, a second algorithm is used to increase k-redundancy. k-redundancy of a node is the minimum number of node removals required to disconnect any two neighbors of that node [50]. It provides a measure to represent the robustness of the network to node failures. The algorithm identifies the minimum number of repairing locations such that all deployed nodes achieve at least m-redundancy. In the experiments m was taken as 2. Then, the robots are commanded to position at those locations.

The experiment was operated with the GUI and several basic functionalities were used including the robot trajectory following. During the diagnosis stage, several mobile robots patrolled cooperatively the area at the same time. The obstacle avoidance basic functionality ensured safe multi-robot coordination. The programs used in the experiments followed an hybrid centralized/distributed approach. Each robot created its connectivity matrix and transmitted it to a central program which merged it, identified the repairing locations and allocated repairing locations to the robots. A video of one of the experiments carried out is shown in [51].

Relocation of nodes is useful when the damage of the network does not compromise the mission, for instance in case of incorrect or non-optimal location of deployed nodes. The testbed can also be suitable for these experiments. The addition of robotic arms and perception tools to allow accurate WSN node retrieving is object of current development.

In the previous experiment the robot helped the WSN. The testbed also supports experiments where static nodes helped the robots. That is the case of HANSEL algorithm [52]. HANSEL is distributed routing method for guiding mobile robots in a passive wireless environment. The robot can detect items that are in its proximity, but items cannot communicate directly with each other. In a learning stage, while searching for a specific node, the robots populate the memory of the nodes they encounter with information about the nodes they have already seen. Later, when asked by robots, the static nodes give the local directions they should take to reach their final destination. The robot motion basic functionalities, except collision avoidance, were substituted by the HANSEL guiding. The node antennas were shielded and their transmission power was reduced to the minimum in order to simulate the passive wireless environment. The location and orientation of the robots were monitored with the GUI. IP cameras provided general views. A video of one of the experiment is shown in [53].

### 6.2. Cooperative Localization and Tracking of Mobile Objects

Localization in indoors or in GPS-denied environments is an active research field. Due to the repeatability of robots motion and the ground truth localization, the testbed is suitable to evaluate and compare their performance. Several methods have been experimented using diverse sensors and approaches.

**Figure 8.**
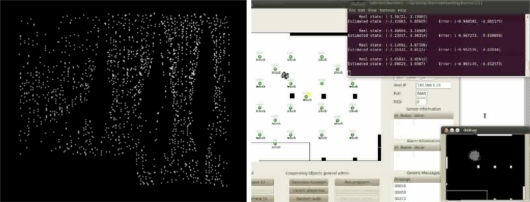
(Left) RSSI raw measurements map for node *n20*; (Right) Snapshot showing the particles estimated location and real robot location during a remote experiment.

The testbed has been used in range-based localization and tracking using RSSI measurements from static WSN nodes. In indoors, due to reflections and other interactions with the environment, it is very difficult to derive a general model for the relation between RSSI measurements and distance. The method tested was based on previously registered RSSI maps. The robot patrols the environment and periodically broadcasts beacon messages. The static nodes receive the messages and respond. The RSSI from each static node is measured and registered. Figure 8 left shows the RSSI raw measurements corresponding to node *n20*, located at the right top of the testbed room. Brighter colors represent higher radio strength. With these measurements RSSI maps are generated and later, they are used for localization. The robot measures RSSI from the static nodes and estimates its location using a Particle Filter (PF) [54] taking into account the similarities with the RSSI map. The robot estimated location is computed as the weighted average of the PF particles.

This experiment was executed remotely. The robot motion was specified using the waypoint following functionalities. The user developed programs for the WSN static nodes, for the WSN nodes attached to each robot and the PF-based localization program running on each robot processor. During the debugging process the algorithm was executed remotely on the user PC, as the Remote User Program depicted in Figure 7. The experiment was monitored on-line with the GUI and the IP cameras. Figure 8 right shows results from one of the experiments.

**Figure 9.**
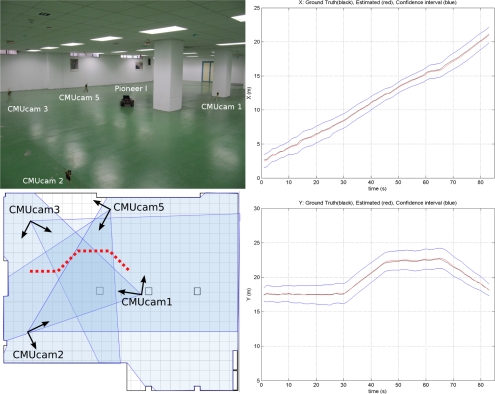
(Left) Object tracking experiment using 5 CMUcam3 cameras; (Right) Results.

The testbed has also been used for localization and tracking using CMUcam3 modules mounted on static WSN nodes. A partially distributed approach was adopted. Image segmentation was applied locally at each WSN camera node. The output of each WSN camera node, the location of the objects segmented on the image plane, is sent to a central WSN node for sensor fusion using an Extended Information Filter (EIF) [55]. All the processing was implemented in TelosB WSN nodes at 2 frames per second. This experiment makes extensive use of the WSN-Player interface for communication with the CMUcam3. Figure 9 shows one picture and the results obtained for axis X (left) and Y (right) in one experiment. The ground truth is represented in black color; the estimated object locations, in red and; the estimated 3*σ* confidence interval is in blue.

### 6.3. Active Perception

The objective of active perception is to perform actions balancing the cost of the actuation and the information gain that is expected from the new measurements. In the testbed actuations can involve sensory actions, such as activation/deactivation of one sensor, or actuations over the robot motion. In most active perception strategies, the selection of the actions involves information reward *versus* cost analyses. In the so-called greedy algorithms the objective is to decide which is the next best action to be carried out, without taking into account long-term goals. Partially Observable Markov Decision Processes (POMDP) [56], on the other hand, consider the long-term goals providing a way to model the interactions of platforms and its sensors in an environment, both of them uncertain. POMDP can tackle rather elaborate scenarios. Both types of approaches have been experimented in the testbed.

**Figure 10.**
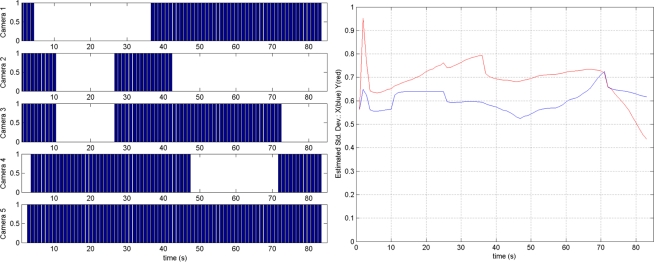
Results in an experiment of active object tracking with CMUcam3 modules.

A greedy algorithm was adopted for the cooperative localization and tracking using CMUcam3. At each time step, the strategy activates or deactivates CMUcam3 modules. In this analysis the cost is the energy consumed by an active camera. The reward is the information gain about the target location due to the new observation, measured as a reduction in the Shannon entropy [57]. An action is advantageous if the reward is higher than the cost. At each time the most advantageous action is selected. This active perception method can be easily incorporated within a Bayesian Recursive Filter. The greedy algorithm was efficiently implemented in the testbed WSN nodes. Figure 10 shows some experimental results with five CMUcam3 cameras. Figure 10 left shows which camera nodes are active at each time. Camera 5 is the most informative one and is active during the whole experiment. In this experiment the mean errors achieved by the active perception method were almost as good as those achieved by the EIF with 5 cameras (0.24 *versus* 0.18) but they needed 39.49% less measurements.

Logging of images from the CMUcam3 is possible using its inner MMC/SD card. In this case, the testbed engineer should manually download the images and make them available to the user.

POMDP for mobile object-target tracking using a set of robots-pursuers were also experimented in the CONET testbed. The pursuer robots were equipped with sensors to detect the target. POMPDs were used to decide which of the possible robot actions (turn left, right, stay, go forward) is the optimum to cooperatively reduce target uncertainty. This experiment deals with POMPDs scalability by adopting a decentralized scheme including decentralized data fusion for coordinated policy execution and auctioning of policies for robot cooperation. A video of one of the experiments is shown in [58].

### 6.4. Multi-Robot Exploration and Mapping

Exploration in difficult or large environments is an active research field in robotics community. There are several approaches that aim to keep the team of robots connected, but even so communication constraints imposes an inherent limit to the range that can be explored. These experiments examined two approaches: greedy exploration and role-based exploration. In the greedy exploration robots opportunistically seek to expand their knowledge of the world and coordinate with teammates when possible, but there is no effort to relay information. In role-based exploration, the team conforms to a hierarchy with robots exploring the far reaches of the environment and relays acting as mobile messengers, ferrying sensor and mapping information back and forth between base station and explorers [59].

The testbed was used to compare both methods in several conditions. Two types of environments were used: the testbed room with different configurations and obstacles and a larger and more complex environment, the hallways of the building of the School of Engineering, with an area with more than 3000 m^2^. Different experiments were carried out involving two and four robots. The experiments showed the superiority of the role-based approach in terms of area explored, exploration time and adaptability to unexpected communication dropout [60]. A video of one experiment is in [61].

The user program was a central planner running in a laptop that communicated to the robots Player Servers. The central planner executed the robot task allocation using both approaches and combined the maps individually generated by each robot. The program at each robot executed commands provided by the central planner using the Wavefront-Propagation path-planner functionality. Also, using the laser measurements, it generated maps of the area surveyed by the robot. The ground truth localization was used in the experiments carried out in the testbed room. In the larger experiments, robot localization was obtained by the AMCL method, also provided as basic functionality. This experiment only involved robotic platforms and illustrates the use of the testbed to evaluate and compare multi-robot algorithms.

## 7. Conclusions

Cooperation between sensors and platforms with heterogeneous capabilities is attracting great interest in academic and industrial communities due to its high possibilities in a large variety of problems. However, the number of experimental platforms for evaluation and comparison of cooperative algorithms remains still low for an adequate development of these technologies. This paper presents a remote testbed for cooperative experiments involving mobile robots and Wireless Sensor Networks (WSN). Robots and WSN have very diverse capabilities. Although making them interoperable is not easy, this diversity is often the origin of interesting synergies.

The testbed is currently available and is deployed in a 500 m^2^ room at the building of the School of Engineering of Seville. It comprises 5 Pioneer 3AT mobile robots and one RC-adapted robot and 4 sets of different WSN nodes models, which can be static or mobile, mounted on the robots or carried by people. These platforms are equipped with the sensors most frequently used in cooperative perception experiments including static and mobile cameras, laser range finders, GPS receivers, accelerometers, temperature sensors, light intensity sensors, among others. The testbed provides tight integration and full interoperability between robots and WSN through a bidirectional protocol with data, request and command messages.

The testbed is open at several levels. It is not focused on any specific application, problem or technology. It can perform only WSN, only multi-robot or robot-WSN cooperative experiments. Also, its modular architecture uses standard tools and abstract interfaces. It allows performing experiments with different levels of decentralization. As a result, the proposed testbed can hold a very wide range of experiments. It can also be remotely operated through a friendly GUI with full control over the experiment. It also includes basic functionalities to help users in the development of their experiments.

The presented testbed has been used in the EU-funded Cooperating Object Network of Excellence CONET to assess techniques from academic and industrial communities. The main experiments already carried out focused on cooperative tracking using different sets of sensors, data fusion, active perception, cooperative exploration and robot-WSN collaboration for network diagnosis and repairing.

This work opens several lines for research. Numerous robots and WSN simulators have been developed. Although some research has been done, the development of simulators involving both systems working in tight cooperation and with full interaction capabilities still needs further research. Such simulators could allow testing the experiment before implementing it in the testbed. Also, hybrid hardware-software testbeds, where some components are hardware while others are simulated, could be interesting tools, particularly in the development and debugging of complex experiments or if some components are not available at that moment. Other current development lines are the extension with a larger number of sensors and platforms, such as smartphones and Unmanned Aerial Vehicles, migration to ROS and the enlargement of the library of basic functionalities.

## Figures and Tables

**Table 1.** Main features of the testbeds reported in the literature.

	Heterogeneous	Flexible and Modular	Open	Easy to Use	Remote Access	Application					
						General	Env. Monitoring	Networking	Surveillance/SAR	Smart Cities	Localization/Mapping
Static WSN testbeds	[19]	[19]	[22]		[19] [21]	[23]	[19]	[20] [22]		[23]	[21]
Mobile robots testbeds	[18]	[13] [15]	[17]	[13]	[15]	[13] [14] [16] [18]		[15]	[12]		[10] [11]
Partially integrated testbeds		[25]	[28]	[28]	[28]	[28]		[25]	[30]		[29] [31]
Highly integrated testbeds	[33] [34]	[33] [32]				[34]				[33] [32]	

**Table 2.** Main features of the sensors mounted on the mobile robots.

**Sensor**	**Physical Magnitude**	**Main specifications**	**Data size (bytes)**	**Power (mW)**	**Qty**
*Microsoft* Kinect	Distance (m) Color (RGB) Infrared image Accelerat. (m/s^2^)	Range 0.4–4 (m) Resolution 640 × 480 (px) FOV (57,43) (deg) Freq. 30 (fps)	922 k(RGB) 422 k(IR)	12	7
*Imaging Source* 21BF04 Camera	Color (RGB) Light (intensity)	Resolution 640 × 480(px) Freq. 60 (fps)	922 k(RGB) 307 k(BW)	2.4	6
*Hokuyo* UTM-30LX	Distance (m)	Range 0.1–30 (m) FOV 270 (deg) Accuracy ±3 (cm) Resolution 0.25 (deg)	1,080	8	7
MC-1513 GPS	Longitude (deg) Latitude (deg) Altitude (m) Velocity (m/s)	Accuracy 2.5 (m) Freq. 10 (Hz) Range 0–18,000 (m) Range 0–515 (m/s)	37	0.2	7
Ez-compass-3A	Angle (deg) Mag.F. (Gauss) Accel. (*m/s*^2^)	Accuracy 0.5 (deg) Resolution 0.08 (deg), 1(mGauss) Freq. 10 (hz)	53	0.54	2

**Table 3.** Main features of WSN sensors.

**Sensor**	**Physical Magnitude**	**Main specifications**	**Data size (bytes)**	**Power (mW)**	**Qty**
CMUcam2/3	Color image	Resolution 352 × 288 (px) Freq. 1 (fps)	304,128	650	9
*Hamamatsu* S1087	Light intensity Infrared intensity	Spectral range 320–730 (nm) Spectral range 320–1,100 (nm)	2	<1	30
*Sensirion* SHT11	Humidity (%) Temperature(°C)	Range 0–100 (%), [−40,123.8] (°C) Resolution 0.03 (%), 0.01 (°C) Accuracy ±3.5 (%), (25 °C)	2	<1	30
*Honeywell* HMC1002	Magnetic Field (gauss)	Range ±2 Resolution 27 (*μ*gauss)	2	20	30
*Panasonic* ERTJ1VR103J	Temperature(°C)	Range [−40, 70] (°C)	2	<1	10
CdSe photocell	Light intensity	Maximum Sensitivity 690 (nm) Range 2–520 (kΩ)	2	<1	10
ADXL202JE 2-axis Accelerometer	Acceleration (m/s^2^)	Range ±2 (g) Resolution 2 (mG) Sensitivity 167 (mV/G) ± 17% Accuracy 2 (mg) (60 Hz) Zero g bias 2 (mg/°C) (25 °C)	4	<1	8
Panasonic WM62A Microphone	Sound Freq. (Hz) Amplitude (V)	Range 20–16,000 (Hz) Sensitivity −45 ± 4 dB Impedance <2.2 kΩ	2	<1	5
*Intersema* MS5534AM	Pressure (mbar)	Range 300–1,100 Resolution 0.01 Accuracy ±1.5% (25 °C)	2	<1	5
*TAOS* TSL2550D	Light intensity	Spectral range 400–1,000 (nm)	2	<1	5

**Table 4.** Examples of messages in the robot-WSN interface.

**type**	**routing header**		**data**			
SENSOR DATA	CO ID	Parent ID	number of sensors	type 1	type 2	type N
				value 1	value 2	value N
COMMAND	CO ID	Parent ID	command type	param. size	parameter 1	parameter N
POSITION	CO ID	Parent ID	X	Y	Z	state
USER DATA	CO ID	Parent ID	data size	byte 1	byte 2	byte N
